# Effect of saturated and unsaturated fatty acid supplementation on bio-plastic production under submerged fermentation

**DOI:** 10.1007/s13205-012-0110-4

**Published:** 2013-01-18

**Authors:** S. K. Srivastava, Abhishek Dutt Tripathi

**Affiliations:** School of Biochemical Engineering, Institute of Technology, Banaras Hindu University, Varanasi, 221005 India

**Keywords:** Polyhydroxyalkanoates (PHAs), Fatty acid supplementation, Bio-plastic, Characterization

## Abstract

Polyhydroxyalkanoates (PHAs) are intracellular reserve material stored by gram-negative bacteria under nutrient-limited condition. PHAs are utilized in biodegradable plastics (bio-plastics) synthesis due to their similarity with conventional synthetic plastic. In the present study, the effect of addition of saturated and unsaturated fatty acids (palmitic acid, stearic acid, oleic acid and linoleic acid) on the production of PHAs by the soil bacterium *Alcaligenes* sp. NCIM 5085 was studied. Fatty acid supplementation in basal media produced saturated and unsaturated PHAs of medium and short chain length. Gas chromatography analysis of palmitic acid-supplemented media showed the presence of short chain length (scl) PHAs which could potentially serve as precursors for bio-plastic production. The scl PHA was subsequently characterized as PHB by NMR and FTIR. On the other hand, oleic acid and linoleic acid addition showed both saturated and unsaturated PHAs of different chain lengths. Palmitic acid showed maximum PHB content of 70.8 % at concentration of 15 g l^−1^ under shake flask cultivation. When shake flask cultivation was scaled up in a 7.5-l bioreactor (working volume 3 l), 7.6 g l^−1^ PHA was produced with a PHB yield (*Y*_P/X_) and productivity of 75.89 % and 0.14 g l^−1 ^h, respectively.

## Introduction

Polyhydroxyalkanoates (PHAs) are polymers of hydroxyalkanoate that are synthesized and accumulated as intracellular carbon and energy storage in various microorganisms. In comparison to the conventional petroleum-derived plastics, PHAs are attracting considerable attention in the plastic market due to their biodegradability under aerobic/anaerobic conditions, enormous application in the field of tissue engineering and as environment friendly packaging material (Liu et al. 2007). PHA composition is profoundly affected by the type of carbon source and the monomeric composition of PHA determines the quality of bio-plastic. Fatty acids can be used as supplements in the formation of PHA with monomers having the potential to serve as biodegradable plastics. Fatty acid β-oxidation leads to increase in the acetyl-CoA concentration which in turn shifts the TCA cycle to PHA synthesis. Hence, the present study explores the effect of fatty acids as nutritional supplement on PHA production by an *Alcaligenes* sp. Oleic and lauric acid have been extensively used as nutritional supplements for PHA production in *Aeromonas hydrophila* (Chen et al. 2001), *Ralstonia eutropha* (Marangoni et al. 2000), recombinant *Escherichia coli XY1*-*Blue pSYL105* (Lee et al. 1995). Certain vegetable oil supplementation in basal media enhanced exopolysaccharide production (Bolla et al. 2011; Park et al. 2001). It has also been shown that plant and vegetable oil produce a stimulatory effect on the production of polysaccharides (Yang et al. 2000; Huisman et al. 1989), mcl PHA synthesis (Gustavo and Regina 2006).

In the present research, efforts have been made to study the effects of saturated and unsaturated fatty acid supplementation on biodegradable plastic production by the soil bacterium *Alcaligenes* sp. in a submerged fermentation process. In addition, the effect of these additives on PHA content was also investigated. Optimization of fatty acid concentration for enhanced PHA production was also performed. PHA produced in shake flask cultivation was scaled up in a bioreactor. The characterization of PHA was performed by Fourier transform infrared spectroscopy (FTIR) and NMR.

## Materials and methods

### Bacterial strain

*Alcaligenes* sp. NCIM No. 5085 was obtained from National Chemical Laboratory (NCL), Pune, India.

### Growth and production media

The growth medium, mineral salt medium (MSM), contained (g l^−1^): fructose 10, urea 0.8, KH_2_PO_4_ 2.0, Na_2_HPO_4_ 0.6, MgSO_4_·7H_2_O 1.0, yeast extract 0.1 and 1 ml l^−1^ of trace element [ZnSO_4_·7H_2_O 1.3, CaCl_2_ 20.0, FeSO_4_·7H_2_O 0.2, (NH_4_)_6_Mo_7_O_24_·4H_2_O 0.6 and H_3_BO_3_ 0.6]. Fructose was sterilized separately at 121 °C for 15 min and added aseptically into the flask containing the other components at room temperature. The pH of the final culture medium was adjusted to 7 ± 0.5 with 0.1 N HCl or 0.1 N NaOH prior to inoculation. Production media was prepared in 250 ml conical flask containing 100 ml MSM. Composition of production media was same as growth media, except that fructose concentration in production media was 40 g l^−1^. Five ml of the seed of each bacterial strain inoculum was added into different conical flasks containing 100 ml of production medium and incubated at 150 rpm, 37 °C for 48 h. Samples were extracted for PHA analysis at different time intervals, viz, 12, 24, 36, 48 and 72 h, respectively.

### Fatty acid supplementation

Four types of fatty acids: palmitic acid (16:0), stearic acid (18:0), oleic acid (18:1) and linoleic acid (18:2) were used as nutritional supplements at a concentration of 5 g l^−1^ for studying PHA production. All the experiments were performed in triplicates.

## Analytical study

### Dry cell mass

Broth culture of 20 ml was centrifuged at 10,000 rpm for 10 min at 4 °C, the cell pellet washed with saline water (NaCl 0.8 %, wt vol^−1^) and dried in aluminium weighing dishes at 90 °C for 24 h. This dry cell mass was further used for PHA extraction and estimation.

### PHA extraction

PHA was extracted using chloroform–hypochlorite extraction method. Pure PHA was obtained by non-solvent precipitation (five times the volume of chloroform) and filtration. The non-solvent used was a mixture of methanol and water (7:3, vol vol^−1^). Filtration was performed using membrane filters (2 μm, Millipore).

### PHA estimation

Samples for GC analysis were prepared as described by Braunegg et al. (1978). Analysis was performed in a GC (Shimadzu, Model: QP-5000) equipped with a flame ionization detector (FID) with a split less injection (80:1) using a DB wax column (polar, 30 m, 0.32 μm, 0.25 μm thickness). The carrier gas used was nitrogen at flow rate of 10 ml min^−1^, the injector temperature was maintained at 250 °C, the oven temperature was set at 50 °C which was increased to 200 °C at the rate of 15 °C min^−1^ for 30 min. Benzoic acid was used as internal standard.

### FTIR and NMR analysis

Sample preparation for Fourier transform infrared spectroscopy (FTIR) was performed in a Bruker model IFS-55 FTIR spectrometer coupled to a Bruker IR microscope fitted with an IBM compatible PC running OPUS, Version 2.2 software (Tripathi and Srivastava 2011). ^1^H NMR spectra was obtained at 500 Hz with Bruker Advance 500 in chloroform-d at 45 °C.

## Results and discussion

### Effect of saturated fatty acid supplementation on PHA composition

**Fig. 1:**
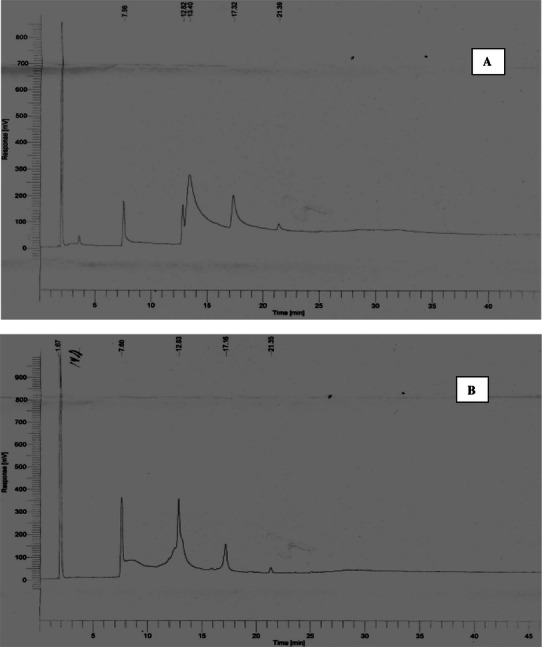

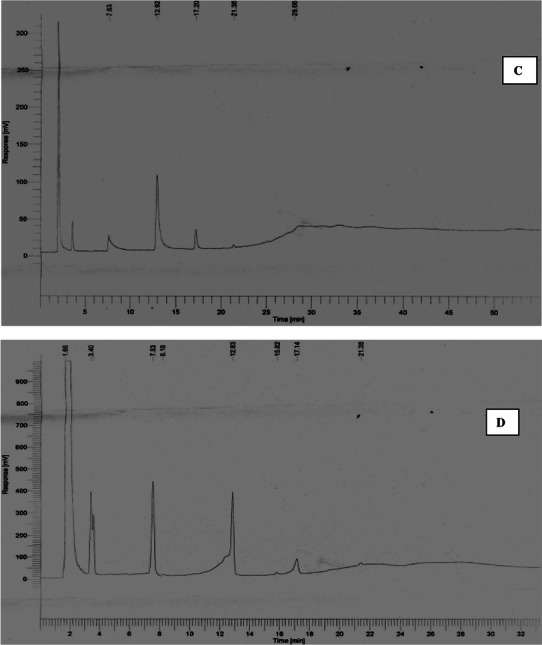
Gas chromatography (GC) analysis of PHA extracted from *Alcaligenes* sp. on basal media for PHA production in shake flask cultivation **a** non-supplemented media, **b** palmitic acid (5.0 g l^−1^)-supplemented media, **c** oleic acid (5.0 g l^−1^)-supplemented media and **d** linoleic acid (5.0 g l^−1^)-supplemented media

Effect of fatty acid supplementation on PHA yield and its composition were studied by collecting 10 ml of samples from different trials after 60.0 h of cultivation. PHA recovered from bacterial strain under different cultivation conditions was analyzed by GC. Gas chromatogram of PHA extracted from *Alcaligenes* sp. grown on MSM after 60.0 h of cultivation showed short chain length (scl) and medium chain length (mcl) PHAs, viz, 3-hydroxybutyrate (3HB); 3-hydroxydocanoate (3HD); 3-hydroxy 9 carbon saturated alkanoate (C9:0) and 3-hydroxy 12 carbon saturated alkanoate (C12:0) with retention time (RT) of 7.60, 12.83, 17.16 and 21.25 min, respectively (Fig. 1a). Table 1 depicts the effect of fatty acid supplementation on monomer composition of PHA. PHA produced on MSM containing fructose as sole carbon source constituted 56.80 % 3HB, 36.10 % 3-hydroxydodocanoate (3HDD), 5.60 % 3-OH saturated alkanoate (9:0) and 1.0 % 3-OH saturated alkanoate (12:0). Trace amount (<0.5 mol% PHA) of 3HV was also produced from *Alcaligenes* sp. grown on MSM.

**Table 1:** Cell and PHA yield grown with fatty acid supplementation

Fatty acid (5 g l^−1^)	Cell dry weight (CDW, g l^−1^)	PHA content (% CDW)	Monomer composition (mol%)													
			3HP	3HB	3HV	3HH	3HO	3HD	3HDD	3HD∆^6^	3HD∆^5^	3-OH (9:0)	3-OH (12:0)	4CL	4BL	3-OH (14:1)
Basal media	3.40 ± 0.10	54.23 ± 0.61	ND	56.80	Tc	ND	ND	ND	36.1	ND	ND	5.60	1.00	ND	ND	ND
Palmitic acid (16:0)	4.12 ± 0.04	70.8 ± 0.08	ND	65.49	Tc	Tc	Tc	ND	19.56	ND	ND	ND	0.5	12.45	Tc	ND
Steric acid (18:0)	4.67 ± 0.05	58.42 ± 0.69	ND	58.89	Tc	Tc	Tc	ND	Tc	18.98	8.33	10.38	1.42	ND	Tc	ND
Oleic acid (18:1)	4.90 ± 0.04	67.39 ± 0.31	1.45	63.67	10.36	0.5	Tc	Tc	ND	ND	10.23	Tc	Tc	Tc	ND	11.24
Linoleic acid (18:2)	3.87 ± 0.03	61.67 ± 0.32	ND	56.65	Tc	Tc	14.56	ND	10.23	20.87	Tc	Tc	ND	15.78	0.5	6.36

Results are presented as mean values ± SD for triplicate analysis

3HP, 3-hydroxyproponoate; 3HB, 3-hydroxybutyrate; 3HV, 3-hydroxyvalerate; 3HH, 3-hydroxyhexanoate; 3HD, 3-hydroxydodecanoate; 3HDD, 3-hydroxydodecanoate; 3-HD∆^6^, 3-hydroxydocanoate (1 double bond at 6th carbon); 3-HD∆^5^, 3-hydroxydocanoate (1 double bond at 5th carbon); 3-OH (9:0), 3-hydroxy saturated alkanoate (9 carbon chain); 3-OH (12:0), 3-hydroxy 12 carbon saturated alkanoate; 4CL, 4-caprolactone; 4BL, 4-butyrolactone; 3-OH (14:1), 3-hydroxy 14 carbon unsaturated alkanoate; ND, not detected; Tc, trace amount <0.5 %

Palmitic acid (16:0) incorporation in basal MSM gave scl PHA including PHB and mcl PHAs consisting of 3HD, 4-caprolactone (4CL, RT 17.19 min), 12 carbon 3-hydroxyalkanoate (RT 21.35 min) and 14 carbon 3-hydroxyalkanoate (RT 28.06 min) (Fig. 1b). Palmitic acid addition in MSM enhanced PHA yield from 54.23 ± 0.61 to 70.8 ± 0.08 %, which is in correlation with a previous finding (Lo et al. 2005). 3HB constituted 65.49 % of PHA (% mol) in palmitic acid-supplemented media. MCL PHAs, 3HDD and 4CL also constituted PHA which is in conformity with a previous finding (Eggink et al. 1990; Huisman et al. 1989). Formation of different monomers on the same substrate indicates that a common intermediate of fatty acid metabolism serves as precursor in the synthesis of PHA monomers. Acetyl-CoA is a likely candidate for this because of its central role in the carbohydrate metabolism.

Gas chromatogram (GC) of stearic acid-supplemented media produced scl and mcl PHAs, viz., 3HB, 3HD∆^6^, 3HD∆^5^, 3-OH (9:0) and 3-OH (12:0) saturated alkanoate (figure not shown). The monomers 3HD∆^5^ and 3HD∆^6^ exactly match the acyl moieties of 3-hydroxy-acyl carrier protein intermediates of the unsaturated fatty acid biosynthetic pathway. Stearic acid (18:0) addition in MSM increased cell mass from 3.40 to 4.67 g l^−1^. However, PHA content was lesser in comparison to PHA content observed in MSM supplemented with palmitic acid. This may be attributed to higher specificity of PHA synthase toward palmitic acid breakdown intermediates in comparison to stearic acid.

PHA produced on MSM supplemented with stearic acid comprised 3HB, 3HD∆^6^, 3HD∆^5^ and 3-OH (9:0) saturated alkanoate at molar composition of 58.89, 18.98, 8.33 and 10.38 %, respectively (Table 1).

### Effect of unsaturated fatty acid supplementation on PHA composition

Oleic acid (18:1) and linoleic acid (18:2) addition in MSM for PHA production increased mcl saturated and unsaturated hydroxyalkanoates (Fig. 1c, d). It can be clearly elucidated from Fig. 1c that oleic acid addition in PHA cultivation media produced 3-hydroxyvalerate (3HV) and 3HD∆^5^ along with 3HB. Monomeric composition of PHA showed that it contained 63.67 % 3HB, 11.24 % 3-OH (14:1) unsaturated alkanoate, 10.36 % 3HV and 10.23 % 3HD∆^5^ (Table 1) which is similar to a previous finding (Braungg 1994). *Alcaligenes* sp. showed higher PHA yield under similar condition in comparison to a previous report (Satish et al. 2011) due to enhanced PHA synthase and reduced depolymerase activity. PHAs formed from euphorbia oil comprised similar monomers generated via β-oxidation of vernolic acid (δ12, 13-epoxy-9c-octadecenoic acid), the main component of euphorbia oil (Eggink et al. 1995).

Linoleic acid (18:2)-supplemented basal media produced 61.67 ± 0.32 % PHA with 3HB content of 56.65 % (% mol PHA). Linoleic acid addition in MSM gave wide range of mcl saturated, ringed and unsaturated hydroxyalkanoates having 6–20 carbon chain length, viz, 3HO, 3HDD, 3HD∆^6^, 4CL, 4-butyrolactone and 3-OH (14:1) unsaturated alkanoate. 3HDD and 3HD∆^6^ monomers are produced via three cycles of β-oxidation. The synthesis of both saturated and unsaturated PHA monomers by linoleic acid addition in MSM may be attributed to generation of sequential intermediates in the fatty acid metabolic pathway of bacteria suggesting a possible linkage between de novo fatty acid oxidation and PHA synthesis (Slater et al. 1988).

Oleic acid and linoleic acid showed pronounced effect on cell mass. Oleic acid supplementation enhanced cell mass; however, linoleic acid addition showed lesser cell mass in comparison to non-fatty acid-supplemented MSM (Table 1). This may be attributed to the presence of two double bonds in linoleic acid which suppressed the cell growth. The monomers 3HD∆^5^ and 3HD∆^6^ exactly match the acyl moieties of 3-hydroxyacyl-acyl carrier protein intermediates of the unsaturated fatty acid biosynthetic pathway. Previously, *Pseudomonas* *putida* grown in oleic acid- and linoleic acid-supplemented medium showed similar PHA monomers which are formed though a common intermediate 3-hydroxyacyl-CoAs, derived from the dienoyl-CoA reductase pathway (Tan et al. 1997).

Palmitic acid increased PHA content (% CDW) and dry cell mass in comparison to stearic acid and unsaturated fatty acids (oleic and linoleic acid) which is in correlation with a previous finding of Lo et al. (2005), who reported that saturated fatty acid (palmitic acid and stearic acid) promoted cell growth in *S. natans*. 3HB content was highest in palmitic acid added media in comparison to oleic acid- and linoleic acid-supplemented medium. This may be attributed to decreased NADH-dependent 2,4-dienoyl-CoA reductase activity which reduces ∆^4^ double bond in linoleic acid oxidation pathway (Table 1).

### Effect of palmitic acid concentration on PHA composition

Effect of palmitic acid concentration on PHA content in *Alcaligenes* sp. was studied at different concentrations, viz, 5, 10, 15, 20 and 25 g l^−1^ (Table 2). Maximum PHA concentration of 74.80 ± 0.34 % (CDW) with maximum 3HB content of 70.89 % was obtained in 15 g l^−1^ palmitic acid-supplemented medium. Further increase in fatty acid concentration enhanced mcl PHA but 3HB concentration was decreased.

**Table 2:** Cell and PHA yield grown with palmitic acid supplementation

Palmitic acid concentration (g l^−1^)	Cell dry weight (CDW, g l^−1^)	PHA content (% CDW)	Monomer composition (mol%)			
			HB	HDD	3-OH (12:0)	4CL
5.0	4.12 ± 0.04	54.23 ± 0.55	65.49	20.56	0.5	13.45
10.0	4.56 ± 0.11	58.42 ± 0.42	67.25	17.56	0.5	14.69
15.0	5.5 ± 0.16	74.80 ± 0.34	70.89	13.5	0.7	14.91
20.0	6.1 ± 0.06	67.39 ± 0.43	62.76	18.78	2.3	16.66
25.0	6.7 ± 0.13	61.67 ± 0.34	58.98	24.18	1.8	14.04

Results are presented as mean values ± SD for triplicate analysis

HB, Hydroxybutyrate; HDD, hydroxydodecanoate; 3-OH (12:0), 3-hydroxy 12 carbon saturated alkanoate; 4CL, 4-caprolactone

### Scale up in 7.5 l bioreactor

**Fig. 2:**
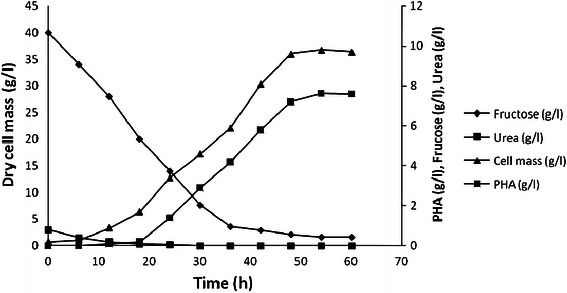
Time profile of PHA and cell mass production, fructose and urea utilization in 7.5 l New Brunswick Bioflo Fermentor (working volume 3.0 l)

Shake flask study was then scaled up to a lab-scale bioreactor. The culture was grown in a 7.5-l bentchtop bioreactor (BioFlo/Celligen 115, New Brunswick, USA) to study PHA production in batch cultivation. Working volume of bioreactor was kept at 3.0 l. Batch cultivation study was carried out to understand the kinetics of PHB production under controlled condition of temperature, pH, agitation and aeration. Figure 2 represents the time profile of PHA synthesis on 15 g l^−1^ palmitic acid-supplemented medium. Fructose and urea served as carbon and nitrogen sources at initial concentrations of 40.0 and 0.8 g l^−1^, respectively. pH was kept at 7.00 ± 0.5 throughout the production process and DO was maintained at 30 % saturation value with an agitation speed of 350 rpm. Agitation speed and DO cascade were done by setting minimum and maximum agitation speed at 100 and 400 rpm, respectively, to maintain the desired dissolved oxygen concentration. Aeration rate during PHB production was kept at 1.5 l min^−1^ and cultivation temperature was set at 37 °C. Figure 2 clearly depicts that after a lag phase of 12.0 h, biomass increased to 9.80 g l^−1^ at 54.0 h. Maximum PHA production was found to be 7.62 g l^−1^ after 54.0 h of fermentative production. Total sugar concentration decreased to 1.6 g l^−1^ at the end of production phase in comparison to initial concentration of 40.0 g l^−1^. Nitrogen source also depleted after 24.0 h of cultivation which leads to enhanced PHB production. PHA yield (*Y*_P/X_) in terms of cell biomass produced and substrate consumed were found to be 0.78 (g g^−1^ cell mass) and 0.27 (g g^−1^ substrate consumed). Percentage PHA yield (*Y*_P/X_) of 78.0 % obtained in the present study is higher than previously reported yield (Tripathi and Srivastava 2011). PHA obtained after scale up on 7.5 l bioreactor comprised 75.89 % HB, 12.34 % HDD, 1.1 % 3-OH (12:0) alkanoate and 10.67 % 4CL. Fed batch cultivation of *P. putida* in 3.0 l bioreactor using oleic acid and nitrogen source produced 44.0 % (% DCW) PHA with % yield of 0.10 (g PHA/g carbon) (Marsudi et al. 2007).

### Characterization of PHA by FTIR and NMR

**Fig. 3:**
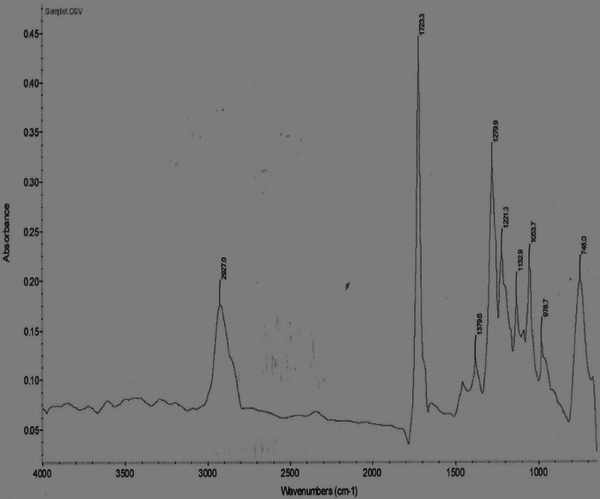
FTIR analysis of PHA extracted from *Alcaligenes* sp. on palmitic acid-supplemented (16 g l^−1^) in basal media for PHA production under optimized cultivation condition

FTIR was used for the evaluation of chemical structure of PHA (Fig. 3). FTIR spectra predicted the presence of functional groups of PHB, i.e., aliphatic C–H, =O stretching, =C–H deformation, =C–H, =CH, =C–O, etc. (Nur et al. 2004). Figure 3 represented a sharp peak at wave number 1,454 and 1,379 cm^−1^ which correspond to the asymmetrical deformation of C–H bond in CH_2_ and CH_3_ groups. The band at 1,724 and 1,279 cm^−1^ corresponded to the stretching of the C=O bond, whereas a series of intense bands located at 1,000–1,300 cm^−1^ reads the stretching of the C–O bond of the ester group. PHB produced by *Alcaligenes* sp. showed a strong adsorption band at 1,279 cm^−1^ which is characteristic for ester bonding and is similar to the absorption band of commercial PHB.

**Fig. 4:**
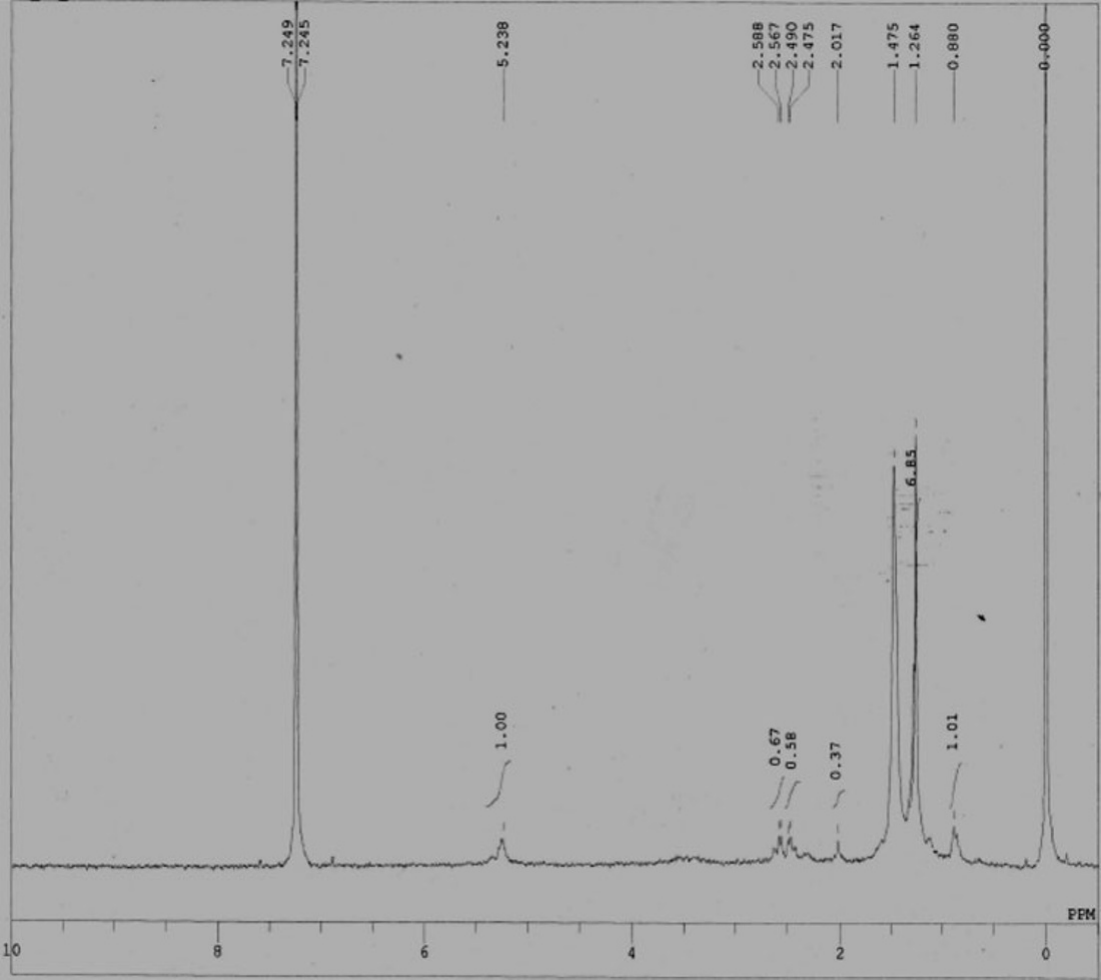
^1^H-NMR (D_2_O) of polyhydroxyalkanoate extracted from *Alcaligenes* sp. in palmitic acid-supplemented media

To reveal the chemical structure of PHB, NMR analysis was done. Figure 4 represented the ^1^H spectra of PHB recovered from *Alcaligenes* sp. in MSM supplemented with 15 g l^−1^ palmitic acid. Multiplet signal obtained at δ5.26 ppm corresponds to CH, doublet at δ2.52 ppm corresponds to CH_2_ and singlet at δ1.274 represents CH_3_ group which is in correlation with a previous finding (Reddy et al. 2010).

In the present study, soil bacterium *Alcaligenes* sp. showed maximum PHA concentration of 70.8 % (% CDW) by supplementing palmitic acid in MSM at a concentration of 15 g l^−1^. Shake flask cultivation scale up in 7.5 l bioreactor (working volume 3.0 l) gave a PHB yield of 78.0 % (DCW) with productivity of 0.14 g l^−1 ^h. The present study clearly suggests that *Alcaligenes* sp. gave maximum PHA yield and productivity in lesser time as reported in previous studies. PHA monomers are derived from β-oxidation of long chain fatty acid but still further genetic and enzymatic studies are needed to elucidate the relationship between fatty acid metabolism and PHA biosynthesis.

## References

[CR1] Bolla K, Hima SVSSL, Bindu N, Samatha B, Singara Charya MA (2011). Effect of plant oils, surfactants and organic acids on the production of mycelial biomass and exopolysaccharides of *Trametes* spp. J Agric Technol.

[CR2] Braunegg G, Sonnleitner B, Lafferty RM (1978). A rapid gas chromatographic method for the determination of poly-β-hydroxybutyric acid in microbial biomass. Eur J Appl Microbiol Biotechnol.

[CR3] Braungg G (1994). Gas chromatographic analysis of polyhydroxyalkanoates in bacteria. Biotechnol Tech.

[CR4] Chen GQ, Zhang G, Park SJ, Lee SY (2001). Industrial production of poly(hydroxybutyrate-co-hydroxyhexanoate). Appl Microbiol Biotechnol.

[CR5] Eggink G, Van der Wal H, Huberts GNM, Dawes EA (1990). Production of poly-3-hydroxyalkanoates by *P. putida* during growth on long-chain fatty acids. Novel biodegradable microbial polymers. NATO ASI Series.

[CR6] Eggink G, Waard D, Huijberts P (1995). Formation of novel poly(hydroxyalkanoates) from long chin fatty acids. Can J Microbiol.

[CR7] Gustavo GF, Regina VA (2006). Use of vegetable oils as substrates for medium-chain-length polyhydroxyalkanoates production by recombinant *Escherichia coli*. Biotechnol.

[CR9] Huisman GW, De Leeuw O, Eggink G, Witholt B (1989). Synthesis of poly-3-hydroxyalkanoates is a common feature of fluorescent pseudomonads. Appl Environ Microbiol.

[CR10] Lee SY, Kang SH, Choi CY (1995). Poly(hydroxybutyrate-co-hydroxyvalerate) from glucose and valerate in *Alcaligenes eutrophus*. J Ferment Bioeng.

[CR11] Liu Q, Ouyang SP, Chung A, Wu Q, Chen GQ (2007). Microbial production of R-3-hydroxybutyric acid by recombinant *E. coli* harboring genes of phbA, phbB, and tesB. Appl Microbiol Biotechnol.

[CR12] Lo KW, Chua H, Lawford H, Lo WH, Peter H, Yu F (2005). Effects of fatty acids on growth and poly-3-hydroxybutyrate production in bacteria. Appl Biochem Biotechnol.

[CR13] Marangoni C, Furigo AJR, de Aragao GMF (2000). Oleic acid improves poly(3-hydroxybutyrate-co-3-hydroxyvalerate) production by *Ralstonia eutropha* in inverted sugar and propionic acid. Biotechnol Lett.

[CR14] Marsudi S, Tan IKP, Gan S-N, Ramachandran KB (2007). Production of medium chain length polyhydroxyalkanoates from oleic acid using *Pseudomonas putida* pga1 by fed batch culture. Makara Technol.

[CR15] Nur ZY, Belma A, Yavuz B, Nazime M (2004). Effect of carbon and nitrogen sources and incubation time on poly-beta-hydroxybutyrate (PHB) synthesis by *Bacillus megaterium* 12. Afr J Biotechnol.

[CR16] Park JP, Kim SW, Hwang HJ, Yun JW (2001). Optimization of submerged culture conditions for the mycelial growth and exo-biopolymer production by *Cordyceps militaris*. Lett Appl Microbiol.

[CR17] Reddy VS, Thirumala M, Mahmood M (2010). Production and characterization of PHB from two novel strains of *Bacillus* sp. isolated from soil and activated sludge. J Ind Microbiol Technol.

[CR18] Satish M, Anbuselvi S, Vikram M, Soujanya M (2011). Biosynthesis and characterization of biodegradable plastics from *Pseudomonas oleovorans* and *Alcaligenes eutrophus*. Int J Adv Biotechnol Res.

[CR20] Slater SC, Voige WH, Dennis DE (1988). Cloning and expression in *Escherichia coli* of the *Alcaligenes eutrophus* H16 poly-*p*-hydroxybutyrate biosynthetic pathway. J Bacteriol.

[CR21] Tan IKP, Sudesh Kumar K, Theanmalar M, Gan SN, Gordon B (1997). Saponified palm kernel oil and its major free fatty acids as carbon substrates for the production of polyhydroxyalkanoates in *Pseudomonas putida* PGA1. Appl Microbiol Biotechnol.

[CR22] Tripathi AD, Srivastava SK (2011). Kinetic study of biopolymer (PHB) synthesis in *Alcaligenes* sp. in submerged fermentation process using TEM. J Polym Sci Environ.

[CR23] Yang FC, Ke YF, Kuo SS (2000). Effect of fatty acids on the mycelia growth and polysaccharide formation by *Ganoderma lucidum* in shake flask cultures. Enzyme Microbiol Technol.

